# Strategies to assess the validity of recommendations: a study protocol

**DOI:** 10.1186/1748-5908-8-94

**Published:** 2013-08-22

**Authors:** Laura Martínez García, Andrea Juliana Sanabria, Ignacio Araya, Jennifer Lawson, R Brian Haynes, David Rigau, Ivan Solà, Petra Díaz del Campo, Maria Dolors Estrada, Itziar Etxeandia-Ikobaltzeta, Elvira García Álvarez, Javier Gracia, Anna Kotzeva, Arturo Louro-González, Flavia Salcedo-Fernandez, Maria Mar Trujillo-Martín, Pablo Alonso-Coello

**Affiliations:** 1 Iberoamerican Cochrane Centre- Biomedical Research Institute Sant Pau (IIB Sant Pau), Barcelona, Spain; 2 Evidence Based Dentistry Unit, Faculty of Dentistry, Universidad de Chile, Santiago, Chile; 3 McMaster University, Hamilton, Canada; 4 Health Technology Assessment Unit (UETS), Subdirección General de Tecnología e Innovación Sanitaria, Consejería de Sanidad, Madrid, Spain; 5 Agència de Qualitat i Avaluació Sanitàries de Catalunya, Barcelona, Spain; 6 Osteba, Basque Office for Health Technology Assessment, Vitoria, Spain; 7 Public Health Wales, Locum Consultant in Public Health Medicine, Swansea, UK; 8 Chair of the Scientific Committee of Guíasalud - National Clinical Practice Guideline Programme of the NHS, Madrid, Spain; 9 Centro de Saúde de Cambre, Xerencia de Xestión Integrada de A Coruña, SERGAS, A Coruña, Spain; 10 GuíaSalud-Aragon Institute of Health Sciences, Zaragoza, Spain; 11 Fundación Canaria de Investigación y Salud (FUNCIS), Red de Investigación en Servicios de Salud en Enfermedades Crónicas (REDISSEC), Tenerife, Spain

**Keywords:** Clinical practice guidelines, Diffusion of innovation, Dissemination and implementation, Evidence-based medicine, Information storage and retrieval, Knowledge translation, Methodology, Updating

## Abstract

**Background:**

Clinical practice guidelines (CPGs) become quickly outdated and require a periodic reassessment of evidence research to maintain their validity. However, there is little research about this topic. Our project will provide evidence for some of the most pressing questions in this field: 1) what is the average time for recommendations to become out of date?; 2) what is the comparative performance of two restricted search strategies to evaluate the need to update recommendations?; and 3) what is the feasibility of a more regular monitoring and updating strategy compared to usual practice?. In this protocol we will focus on questions one and two.

**Methods:**

The CPG Development Programme of the Spanish Ministry of Health developed 14 CPGs between 2008 and 2009. We will stratify guidelines by topic and by publication year, and include one CPG by strata.

We will develop a strategy to assess the validity of CPG recommendations, which includes a baseline survey of clinical experts, an update of the original exhaustive literature searches, the identification of key references (reference that trigger a potential recommendation update), and the assessment of the potential changes in each recommendation.

We will run two alternative search strategies to efficiently identify important new evidence: 1) PLUS search based in McMaster Premium LiteratUre Service (PLUS) database; and 2) a Restrictive Search (ReSe) based on the least number of MeSH terms and free text words needed to locate all the references of each original recommendation.

We will perform a survival analysis of recommendations using the Kaplan-Meier method and we will use the log-rank test to analyse differences between survival curves according to the topic, the purpose, the strength of recommendations and the turnover. We will retrieve key references from the exhaustive search and evaluate their presence in the PLUS and ReSe search results.

**Discussion:**

Our project, using a highly structured and transparent methodology, will provide guidance of when recommendations are likely to be at risk of being out of date. We will also assess two novel restrictive search strategies which could reduce the workload without compromising rigour when CPGs developers check for the need of updating.

## Background

Clinical practice guidelines (CPGs) are “statements that include recommendations intended to optimize patient care that are informed by systematic reviews (SRs) of evidence and an assessment of the benefits and harms of alternative care options” [1]. CPGs, just like SRs, become outdated as new evidence is published and require a periodic reassessment of research evidence to remain valid.

Guideline development institutions are concerned about the growing number of CPGs that are not regularly updated [2]. However, methodological handbooks include very little guidance about how to update guidelines other than to do so periodically [3-5]. In general, despite scant research [6], guideline programs endorse three years as a reasonable time period to update their guidelines [7].

Frequently, research in this area focuses on how to identify new evidence. However updating a GCP is a far more complex process and includes three main stages: 1) identifying important new evidence; 2) assess if the new evidence implies the updating of recommendations; and 3) the actual updating.

The identification of important new evidence that justifies an update is a challenge. Usually the original exhaustive search strategy that has been used to identify new evidence to update CPGs is used [8,9]. However, this strategy is very resource intensive, and a barrier to timely updates. Consequently, some studies have evaluated more restricted searches strategies to assess the need to update CPGs [10,11]. These strategies are likely to be sufficient to monitor new evidence and assess the need to update; however, more information is needed about the timing and type of search.

Nowadays, other resources could be used to make the process more efficient [12]. One is the McMaster Premium Literature Service (PLUS) database, from the McMaster Health Knowledge Refinery, which contains a searchable subset of pre-appraised primary studies and SRs from more than 120 journals and since 2003 [13,14]. The PLUS database includes substantially fewer articles than common databases, potentially increasing precision, with a small loss of sensitivity when updating. Recently, PLUS has been shown to be capable of identifying key articles that would be needed to update SRs [15]. These results would suggest that PLUS could prove an efficient method to update CPGs.

### The updating guidelines working group

The Updating Guidelines Working Group goal is to draw on existing work and knowledge in the area of CPGs updating and to provide guidance for both guideline developers and users. Our group has run several studies about CPG updating. We conducted an international survey to identify current practices in CPG updating across guideline development institutions that showed high variability and a lack of standardization of the updating processes [3]. Additionally we conducted a SR that confirmed that there is very limited evidence about what is the optimal strategy or strategies for keeping CPGs up to date [6].

At present we are running several projects to fill this research gap. Our broader project “Assessing the validity and update strategies for CPG: analysis of the GPC National Program for National Health System in Spain” includes three studies addressing three pressing questions in this field: 1) what is the average time for recommendations to become out of date?; 2) what is the comparative performance of two restricted search strategies to evaluate the need to update recommendations?; and 3) what is the feasibility of a more regular monitoring and updating strategy compared to usual practice?.

### Objectives

•Primary objectives:

○Estimate average time for recommendations to become out of date.

○Evaluate two alternative search strategies to assess the validity of CPGs recommendations.

•Secondary objectives:

○Design a strategy to assess the validity of CPGs recommendations.

○Evaluate resources used to perform each strategy.

○Assess the agreement between study participants in identifying references that potentially could update CPGs recommendations.

## Methods

### Design

Intervention study in a cohort of CPGs recommendations.

### Population and eligibility criteria

We will include CPGs developed in the CPG Development Programme of the Spanish Ministry of Health between 2008 and 2009 that are available in English (Additional file 1). We will select a sample of four CPGs. We will stratify guidelines by topic (cancer and palliative care, cardiovascular disease, mental health and metabolic disease) and then by publication year (2008 and 2009). We will select one guide for each topic and two guidelines published in 2008 and two guidelines published in 2009. We will choose the guidelines at random if there is more than one guideline by strata.

### Strategies

We will develop a strategy to assess the validity of recommendations based on the identification (by collating evidence from clinical experts and by exhaustive literature searches) and evaluation of new evidence (Table 1).

**Table 1:** Strategy to assess the validity of recommendations

**Stages**	**What**	**How**	**Who**
Stage 1	Identification of clinical questions and recommendations	Review original CPG	Guideline methodologist from research group
Stage 2	Baseline survey	Recommendation basal survey (http://www.surveymonkey.com)	Clinical experts
Stage 3	Update exhaustive literature search	- Recover original exhaustive literature search	- Guideline methodologist from original CPG
		- Define the filters that will be used: 1) study design; 2) publication date	- Information specialist
Stage 4	References database by clinical questions	Reference management software	Information specialist
Stage 5	First references screening	Reference management software	Guideline methodologist from research group
	- Topic		
	- Study design		
	- Publication type		
Stage 6	References matching	Reference management software	Guideline methodologist from research group
Stage 7	Recommendations database	Statistic program	Guideline methodologist from research group
Stage 8	Second references screening	**Stage 8a** Reference survey to assess the updating effect: feasibility test	- Guideline methodologist from original CPG - Guideline methodologist from research group
	- Identification of relevant references		
	- Identification of key references		
		**Stage 8b** Sample size calculation	Statistician
	- Assess the potential changes in the recommendation		
		**Stage 8c** Reference survey to assess the updating effect (pdf forms)	- Guideline methodologist from original CPG
			- Guideline methodologist from research group
			- Clinical experts
Stage 9	Final report	Final report with study results	Guideline methodologist from research group
	- Recommendations still valid		
	- Recommendation needed update		

*Abbreviations: CPG* Clinical practice guideline.

A PLUS search strategy for the PLUS database (Table 2), and a restrictive search strategy (ReSe) for MEDLINE database (Table 3) will be developed.

**Table 2:** PLUS search strategy

**Stages**	**What**	**How**	**Who**
Stage 1	Identification contents	Review original CPG	Guideline methodologist from research group
Stage 2	PLUS search	- Choose existing MeSH and SNOMED in PLUS database related with original CPG contents	PLUS information specialist
		- Define the filters that will be used: 1) population; 2) study purpose categories; 3) publication date	
Stage 3	Reference databases by CPGs	Reference management software	PLUS information specialist

*Abbreviations: CPG* Clinical practice guideline, *MeSH* Medical Subject Headings, *PLUS* Premium LiteratUre Service, *SNOMED* Systematized Nomenclature of Medicine.

**Table 3:** Restrictive search strategy

**Stages**	**What**	**How**	**Who**
Stage 1	Identification clinical questions	Review original CPG	Guideline methodologist from research group
Stage 2	Clinical questions eligibility	Include clinical questions with ≥ two explicitly PICO components	Guideline methodologist from research group
Stage 3	ReSe	**Stage 3a** Development ReSe serches	Guideline methodologist from research group
		**Stage 3b** Evaluation ReSe serches	
		**Stage 3c** Refinement ReSe serches	
		**Stage 3d** Define the filters that will be used: 1) study design; 2) publication date	
Stage 4	References database by clinical questions	Reference management software	Guideline methodologist from research group

*Abbreviations: CPG* Clinical practice guideline, *PICO* population, intervention, comparator and outcome, ReSe restrictive search.

### Strategy to assess the validity of recommendations

•**Stage 1: Identification of clinical questions and recommendations.** We will extract the clinical questions, the recommendations (identified in the “Summary of recommendations” section) and their strength (SIGN [16] or GRADE [17] system) for each original CPG. Recommendations will be numbered and classified (prevention, screening, diagnosis or treatment).

•**Stage 2: Baseline survey.** Using a similar approach as Shekelle et al. [10] we will conduct a survey by e-mail (http://www.surveymonkey.com) with clinical experts for each CPG. They will evaluate whether they consider that recommendations are up to date and if they know any new studies that might change the recommendations (Additional file 2).

•We will perform the survey in a convenience sample of six clinical experts who participated in the CPG development. Original guideline methodologists will identify the survey participants: 1) four clinical experts representing the different areas covered by the guideline; and 2) two external clinical experts.

•**Stage 3: Update literature search.** We will recover the original exhaustive literature searches per clinical questions.

•Information specialists, preferably from the original guideline, will run the searches in the databases and apply the corresponding study design filters (randomised controlled trials [RCTs] or SRs) used in the original searches. Date filters will be established from the complete year in which the original search was completed onwards.

•**Step 4: References database by clinical question.** We will cluster the references obtained from the baseline survey and from the search. We will identify and eliminate duplicates and build a database by clinical questions with the references identified.

•**Step 5: First reference screening.** We will evaluate whether references are pertinent to the topic of interest, the study design (RCTs or SRs) and the publication type (we will include original articles or abstracts from conferences about original studies) (Additional file 3).

•**Step 6: Reference matching.** We will match pertinent references with one or more related recommendations.

•**Step 7: Recommendations database.** We will analyse the references databases to obtain recommendations: 1) without references; 2) with low turnover (≤ median number of references per recommendation); or 3) with high turnover (> median number of references per recommendation).

•**Step 8: Second reference screening.** We will design a recommendation form to sort out the pertinent references identified (Additional file 4). The form will contain: 1) relative to each recommendation: clinical question, recommendation, evidence quality and strength of recommendation; 2) relative to the related references : citation, ± PubMed Unique Identifier (PMID), abstract and study design; and 3) relative to the assessed references: a question to identify relevant references (references that could be use when considering the update of a recommendation but not necessarily trigger a potential update), a question to identify key references (references that could potentially trigger a recommendation update) and a question to assess the potential changes in the recommendation (in relation with population, intervention, comparison, outcome, quality of evidence, direction and/or strength of the recommendation [18]).

•We will send the recommendations forms to clinical experts and guidelines methodologist by e-mail (we will schedule three remainders every two weeks). Each form will be assessed by two clinical experts and one guideline methodologist. The disagreements will be resolved by consensus.

•**Step 9: Final report.** We will prepare a final report with recommendations that may need updating, in relation to the new evidence identified. The final report will be sent the corresponding institutions that developed these guidelines and the clinicians who will collaborate in the study.

### PLUS search strategy

•**Stage 1: Identification topics.** We will extract the topics for each original CPG (identified in “table of contents”).

•**Stage 2: PLUS search.** PLUS information specialists will develop the corresponding search strategies by matching existing Medical Subject Headings (MeSH) and Systematized Nomenclature of Medicine (SNOMED) with the CPGs topics. They will perform the searches applying PLUS population, study purpose categories (therapy/prevention; diagnosis; prognosis; etiology; economics; clinical predication guide; differential diagnosis) and publication date filters. No filter will be applied to select for either original or review articles.

•**Stage 3: References database by CPGs.** PLUS information specialists will obtain a database of references by CPG.

### Restrictive search strategy

•**Stage 1: Identification of clinical questions.** We will extract the clinical questions for each CPG.

•**Stage 2: Clinical questions eligibility.** Restrictive searches will be structured taking into account the PICO (population, intervention, comparator and outcome) structure of each clinical question. To develop each strategy we will include at least two PICO components from each question and their corresponding most representative keywords. The questions that do not explicitly include PICO components will be excluded.

•E.g. an explicit clinical question from the CPG for Prostate Cancer Treatment is “In patients with prostate specific antigen (PSA) relapse after radical prostatectomy, what kind of salvage intervention is safer and more effective?”. A non explicit clinical question would be “What is the safest treatment and most effective option for a patient with prostate cancer at the locally advanced clinical stage?”. In this question treatment alternatives are not clearly defined and make it a very broad question to be answered by the ReSe strategy [19].

•**Stage 3: ReSe.** To develop ReSe, based on original exhaustive search strategy, we will: 1) Select MeSH terms: If available, for each keyword we will find the most specific MeSH term (e.g. "Prostate-Specific Antigen" MeSH term for the population of the question “In patients with PSA relapse after radical prostatectomy, what kind of salvage intervention is safer and more effective?” [19]); 2) Select free text words [Tw]: for each keyword we will select the most relevant specifics free text words and search them in title (e.g. we would select “prostate[ti] AND specific[ti] AND antigen[ti]” free text words for the question “In patients with PSA relapse after radical prostatectomy, what kind of salvage intervention is safer and more effective?” [19]).

•We will evaluate if the ReSe retrieves all original references considered in the recommendations of the original CPGs. We will evaluate this by calculation the proportion of original references which are retrieved (sensitivity). If a ReSe search does not find all the original references (sensitivity <100%) we will refine it until it retrieves them all.

•For the refinement, if needed, we will be using one or both of the following options: 1) use of less specific MeSH terms; and/or 2) free text words to search in title or abstract. We will limit each ReSe by type of design. For each ReSe we will apply the filter Therapy of the Clinical Study Categories of Clinical Queries, using both narrow and broad scope, and we will apply the SR filter developed at the Health Information Research Unit, McMaster University [20]. Finally, we will perform the searches applying publication date filters.

•**Step 4: References databases by clinical questions.** For each clinical question we will obtain three databases, one using the therapy filter plus narrow scope, one using the therapy filter plus broad scope, and one using the SR filter.

### Outcomes

#### Primary outcomes

•Average time for recommendations to become out of date.

•Proportion references that trigger a potential recommendation update (key references) identified by the alternative search strategies.

#### Secondary outcomes

•Resources used by strategy (time and participants).

•Agreement between clinical experts and guideline methodologists across references screening.

### Analysis

#### Baseline characteristics

We will perform a descriptive analysis of CPGs recommendations included using mean and the standard deviation (for normal distribution), median and range (for abnormal distribution) or absolute and relative frequencies (and the associated 95% CI [confidence interval]), as appropriate.

#### Strategy performance

We will calculate the proportion and 95% CI of pertinent, relevant and key references identified by the exhaustive strategy. We will determine the number of key references from the exhaustive strategy (gold standard) retrieved by PLUS and ReSe strategies. We will estimate the mean time spent on each strategy and the proportion of researchers involved. We will evaluate the agreement between clinical experts and guideline methodologists about the assessment of key references from the exhaustive strategy (step 8). We will calculate the kappa coefficient and the 95% CI, and interpret it according these criteria: poor (0.00-0.20); fair (0.21–0.40); moderate (0.41–0.60); substantial (0.61–0.80); and almost perfect (0.81–1.00) [21].

#### Survival analysis

We will perform a survival analysis of recommendations. We will define the event as the identification of a key reference related to a recommendation. We will consider: 1) recommendation inception date when the original search of each CPG started; 2) recommendation obsolescence date when first key reference is published for potential updated recommendations; and 3) last observation date when the update search of each CPG started for recommendations is still valid. Finally, we will calculate the survival time for the potential updated recommendations (obsolescence date - inception date) and for recommendations still valid (last observation date - inception date).

The estimated rate of survival of recommendations will be calculated using the Kaplan-Meier method and we will use the log-rank test to analyse differences between survival curves according to the topic (cancer, cardiovascular disease, mental health or metabolic disease), the purpose (prevention, screening, diagnosis or treatment), the strength of recommendations and the turnover (number of references linked per recommendation).

#### Sample size

In a feasibility test, we sampled 20.9% (52/249) of recommendations from selected CPGs and identified 17 key references; these warranted an update of eight recommendations (15.4% of recommendations from sample).

Accepting an alpha risk of 0.95 for a precision of ± 0.05 units in a two-sided test for an estimated proportion of 0.154, 112 recommendations randomly selected from the whole recommendations are required assuming that such population corresponds to 249 recommendations. It has been anticipated a replacement rate of 1%.

We will accept p value ≤ 0.05 as significant in all calculations. We will do the analysis with SPSS 18.0 (SPSS Inc., Chicago, Illinois, United States).

## Discussion

In this protocol we are outlining a research project that will address two important questions about the updating of guidelines. Our project will provide evidence both: 1) the assessment of the validity of a cohort of CPGs and; 2) the evaluation of alternative search strategies to update CPGs recommendations.

Using a sample of four CPGs developed in the CPG Development Programme of the Spanish Ministry of Health we will evaluate two potentially more efficient search strategies for the updating of guidelines, and compare them to an exhaustive search strategy (our gold standard). We will include the McMaster Premium LiteratUre Service (PLUS), evaluated for the first time in this context, and an innovative restrictive search strategy. Finally, we will perform a survival analysis of recommendations providing additional evidence about this important topic.

### Our work in the light of previous research

We recently systematically reviewed the research available about strategies for monitoring and updating CPGs [6]. We observed that there is limited evidence about what are the most optimal strategies for this. A restricted search is likely to be sufficient to monitor new evidence and assess the need to update; however, more information is needed about the timing and type of search with only the exhaustive search strategy having been assessed for the actual update of CPGs [6]. The development and evaluation of more efficient strategies is hence needed to improve the timeliness and reduce the burden of maintaining the validity of CPGs.

Only one previous study by Shekelle et al. [10] analysed the survival time of CPGs and suggested that these should be reassessed every three years. We built on the methodology proposed in this study addressing some of its shortcomings. First we will use an exhaustive search strategy, as opposed to the restrictive used by Shekelle et al. [10], that will likely provide a more reliable estimate. We will analyse our results in terms of recommendations out of date, instead of CPGs out of date. Finally, we will also publish a more detailed and explicit approach that will allow developers to be able to implement it in their institutions.

One previous study evaluated the McMaster Premium LiteratUre Service (PLUS) for the updating of SRs with promising results. We therefore decided to include this free of access service as a potential resource that could prove to be highly efficient.

Given all of the above, our research project is timely and fits well with the needs from the guideline community.

### Strengths and limitations

Our study has several strengths. We will use a rigorous and transparent methodology, both to assess the validity of recommendations as well as the performance of the search strategies. We are building on previous research in this area improving its deficiencies [10] and implementing innovative solutions (e.g. standardized reporting) [6]. We will compare three search strategies, head to head, something that only one study, by Gartlehner 2004 et al. [11], has done so far. That study found that the restrictive search (review approach) identified fewer studies but included all-important references rated by their task force. Nevertheless they only evaluated their final strategy in two topics, the results being inconsistent. Finally our group has important expertise in guideline updating [3,6,22] and guideline methodology in general [23,24].

Our study has also some limitations. We will limit our searches by type of study including only SRs and RCTs, however, we think it is unlikely that we will miss important studies that will compromise the generalisability of our findings. Our study will not include the actual updating of the guidelines identified to be out of date and, hence, we will not evaluate whether our strategies are optimal for the final updating. Nevertheless, we believe that our outcome is a reliable surrogate of actual updating.

### Implications of this study

We expect that our work will produce one or more efficient strategies to assess the validity of recommendations, and provide detailed guidance to replicate the process. Furthermore, our results will inform guideline developers about the expected validity of their recommendations in a representative sample of guidelines from a typical cohort of a National Guideline program. If the evaluated search strategies perform optimally, our work could be highly influential for evidence surveillance. Our results could therefore have important implications for a more efficient use of resources in the CPG arena.

## Abbreviations

CI: Confidence interval; CPG: Clinical practice guideline; E.g.: *Exempli gratia*; MeSH: Medical subject headings; PICO: Population, intervention, comparator and outcome; PLUS: Premium literatUre service; PMID: PubMed unique identifier; PSA: Prostate specific antigen; RCT: Randomised controlled trial; ReSe: REstrictive SEarch; SNOMED: Systematized nomenclature of Medicine; SR: Systematic review.

## Competing interests

The authors declare that they have no competing interests.

## Authors’ contributions

LMG, PAC, DR and IS participated in the conception of the study. All authors participated in the design. LMG and PAC drafted a first version of the protocol. All authors participated revising it critically for important intellectual content and have given final approval of the version to be published.

## Supplementary Material

Additional file 1 Clinical practice guidelines developed within the framework of the CPG Development Programme of the Spanish Ministry of Health between 2008 and 2009. Click here for file

Additional file 2 **Recommendation baseline survey to clinical experts.** Example on clinical practice guideline for secondary prevention of stroke (2009) [25]. Click here for file

Additional file 3 Reference screening by pertinence. Click here for file

Additional file 4 **Reference survey to assess the updating effect.** Example on clinical practice guideline for secondary prevention of stroke (2009) [25]. Click here for file

## References

[B1] IOM (Institute of Medicine) Clinical Practice Guideline We Can Trust 2011 Washington, DC: The National Academies Press

[B2] Scottish Intercollegiate Guidelines Network Guidelines by topic [Web] 2001 Edinburgh: Scottish Intercollegiate Guidelines Network - Healthcare Improvement Scotland [last modified 15/04/13; access 22/04/13]. http://www.sign.ac.uk/guidelines/published/index.html

[B3] Alonso-Coello P Martínez García L Carrasco JM Solà I Qureshi S Burgers JS Updating Guidelines Working Group The updating of clinical practice guidelines: insights from an international survey Implement Sci 2011 6 107 10.1186/1748-5908-6-107 21914177 PMC3191352

[B4] Ansari S Rashidian A Guidelines for guidelines: are they up to the task? A comparative assessment of clinical practice guideline development handbooks PLoS One 2012 7 11 e49864 10.1371/journal.pone.0049864 23189167 PMC3506587

[B5] Vernooij R Sanabria AJ Solà I Alonso-Coello P Martínez García L The updating process guidance in clinical practice guidelines handbooks: a systematic review. G-I-N Conference: 10th G-I-N Conference 2013 San Francisco, USA http://www.gin2013.net/ [accepted]

[B6] Martínez García L Arévalo-Rodríguez I Solà I Haynes RB Vandvik PO Alonso-Coello P Updating Guidelines Working Group Strategies for monitoring and updating clinical practice guidelines: a systematic review Implement Sci 2012 7 109 10.1186/1748-5908-7-109 23164220 PMC3520818

[B7] Shekelle P Woolf S Grimshaw JM Schünemann HJ Eccles MP Developing clinical practice guidelines: reviewing, reporting, and publishing guidelines; updating guidelines; and the emerging issues of enhancing guideline implementability and accounting for comorbid conditions in guideline development Implement Sci 2012 7 62 10.1186/1748-5908-7-62 22762242 PMC3503794

[B8] Eccles M Rousseau N Freemantle N Updating evidence-based clinical guidelines J Health Serv Res Policy 2002 7 2 98 103 10.1258/1355819021927746 11934374

[B9] Parmelli E Papini D Moja L Bandieri E Belfiglio M Ciccone G Updating clinical recommendations for breast, colorectal and lung cancer treatments: an opportunity to improve methodology and clinical relevance Ann Oncol 2011 22 1 188 194 10.1093/annonc/mdq324 20605933

[B10] Shekelle PG Ortiz E Rhodes S Validity of the Agency for Healthcare Research and Quality clinical practice guidelines: how quickly do guidelines become outdated? JAMA 2001 286 12 1461 1467 10.1001/jama.286.12.1461 11572738

[B11] Gartlehner G West SL Lohr KN Assessing the need to update prevention guidelines: a comparison of two methods Int J Qual Health Care 2004 16 5 399 406 10.1093/intqhc/mzh081 15375101

[B12] Tsafnat G Dunn A Glasziou P Coiera E The automation of systematic reviews BMJ 2013 346 f139 10.1136/bmj.f139 23305843

[B13] Haynes R Cotoi C Holland J Second-order peer review of the medical literature for clinical practitioners JAMA 2006 295 15 1801 1808 10.1001/jama.295.15.1801 16622142

[B14] Haynes R Holland J Cotoi C McMaster PLUS: a cluster randomized clinical trial of an intervention to accelerate clinical use of evidence-based information from digital libraries JAMIA 2006 13 6 593 600 16929034 10.1197/jamia.M2158 PMC1656957

[B15] Hemens BJ Haynes RB McMaster Premium LiteratUre Service (PLUS) performed well for identifying new studies for updated Cochrane Reviews J Clin Epidemiol 2012 65 1 62 72.e1 10.1016/j.jclinepi.2011.02.010 21856121

[B16] Network SIGN SIGN 50: a guideline developer’s handbook 2011 Edinburgh: SIGN

[B17] Andrews JC Schünemann HJ Oxman AD Pottie K Meerpohl JJ Coello PA GRADE guidelines 15: Going from evidence to recommendation-determinants of a recommendation's direction and strength J Clin Epidemiol 2013 66 7 726 735 10.1016/j.jclinepi.2013.02.003 23570745

[B18] Guyatt GH Oxman AD Kunz R Atkins D Brozek J Vist G GRADE guidelines: 2. Framing the question and deciding on important outcomes J Clin Epidemiol 2011 64 4 395 400 10.1016/j.jclinepi.2010.09.012 21194891

[B19] Working group of the Clinical Practice Guideline on Prostate Cancer Treatment Clinical Practice Guidelines on Prostate Cancer Treatment 2008 Madrid: National Plan for the NHS of the MSC. Aragon Institute of Health Sciences (I+CS) Clinical Practice Guidelines in the NHS I+CS No 2006/02

[B20] Montori VM Wilczynski NL Morgan D Haynes RB Optimal search strategies for retrieving systematic reviews from Medline: analytical survey BMJ 2005 330 7482 68 10.1136/bmj.38336.804167.47 15619601 PMC543864

[B21] Landis JR Koch GG The measurement of observer agreement for categorical data Biometrics 1977 33 159 174 10.2307/2529310 843571

[B22] Grupo de trabajo sobre actualización de GPC Actualización de guías de práctica clínica en el Sistema Nacional de Salud: manual metodológico 2009 Madrid: Ministerio de Ciencia e Innovación

[B23] Alonso-Coello P Irfan A Solà I Gich I Delgado-Noguera M Rigau D Tort S Bonfill X Burgers J Schunemann H The quality of clinical practice guidelines over the last two decades: a systematic review of guideline appraisal studies Qual Saf Health Care 2010 19 1 7 10.1136/qshc.2010.042077 21127089

[B24] Treweek S Oxman AD Alderson P Bossuyt PM Brandt L Brożek J Developing and Evaluating Communication Strategies to Support Informed Decisions and Practice Based on Evidence (DECIDE): protocol and preliminary results Implement Sci 2013 8 6 10.1186/1748-5908-8-6 23302501 PMC3553065

[B25] Development group of the stroke prevention Guideline. Iberoamerican Cochrane Centre, coordinator. Clinical Practice Guideline for Primary and Secondary Prevention of Stroke 2008 Madrid: Quality Plan for the National Health System of the Ministry of Health and Consumer Affairs. Catalan Agency for Health Technology Assessment and Research Clinical Practice Guideline: AATRM Number 2006/15

