# Supplementary material for: Strategies to assess the validity of recommendations: a study protocol
Source: Implement Sci. 2013 Aug 22;8:94. doi: 10.1186/1748-5908-8-94 (PMC3765147; doi:10.1186/1748-5908-8-94)
Supplement: Additional file 1 — Clinical practice guidelines developed within the framework of the CPG Development Programme of the Spanish Ministry of Health between 2008 and 2009. [file 1748-5908-8-94-S1.pdf]

Additional file 1: Clinical Practice Guidelines developed within the framework of the Clinical Practice Guidelines Development Programme of the Spanish Ministry of Health between 2008 and 2009

	Clinical Practice Guideline	Abbreviation	Development Agency	Edition	Topic	Languages[a]
**1**	Clinical Practice Guideline for Treatment of Patients with Anxiety Disorders in Primary Care	Anxiety 2008	Agencia Laín Entralgo	2008	Mental health	Spanish and English version
**2**	Clinical Practice Guideline on the Management of Patients with Autism Spectrum Disorders in Primary Care	Autism 2009	Agencia Laín Entralgo	2009	Mental health	Spanish version
**3**	Clinical Practice Guideline on the Management of Major Depression in Adults	Depression Adult 2008	Avalia-t	2008	Mental health	Spanish and English version
**4**	Clinical Practice Guideline on Major Depression in Childhood and Adolescence	Depression Childhood 2009	Avalia-t	2009	Mental health	Spanish and English version
**5**	Clinical Practice Guideline on Type II Diabetes	Diabetes II 2008	Osteba	2008	Metabolic disease	Spanish version
**6**	Clinical Practice Guideline for Eating Disorders	Eating Disorders 2009	AIAQS	2009	Mental health	Spanish and English version
**7**	Clinical Practice Guideline on the Management of Patients with Insomnia in Primary Care	Insomnia 2009	Agencia Laín Entralgo	2009	Mental health	Spanish version
**8**	Clinical Practice Guideline on Prevention and Treatment of Obesity in Childhood and Adolescence	Obesity 2009	AIAQS CCIb	2009	Metabolic disease	Spanish and English version
**9**	Clinical Practice Guideline on Palliative Care	Palliative Care 2008	Osteba	2008	Cancer and palliative care	Spanish version
**10**	Clinical Practice Guideline for Prostate Cancer Treatment	Prostate Cancer 2008	I+CS	2008	Cancer and palliative care	Spanish and English version
**11**	Clinical Practice Guideline for Schizophrenia and Incipient Psychotic Disorder	Schizophrenia 2009	AIAQS Fòrum de Salut Mental	2009	Mental health	Spanish and English version
**12**	Clinical Practice Guideline for Psychosocial Interventions in Severe Mental Disorder	Severe Mental Disorder 2009	I+CS	2009	Mental health	Spanish version
**13**	Clinical Practice Guideline on the Management of Patients With Stroke in Primary Care	Stroke 2009	Agencia Laín Entralgo	2009	Cardiovascular disease	Spanish version
**14**	Clinical Practice Guideline for Primary and Secondary Prevention of Stroke	Stroke Prevention 2009	AIAQS CCIb	2009	Cardiovascular disease	Spanish and English version

Abbreviations: AIAQS: Agència d'Informació, Avaluació i Qualitat en salut, Cataluña; Agencia Laín Entralgo: Unidad de Evaluación de Tecnologías Sanitarias de la Agencia Laín Entralgo de la Comunidad de Madrid; Avalia-t: Axencia de Avaliación de Tecnoloxías Sanitarias de Galicia; CCIb: Centro Cochrane Iberoamericano; I+CS: Instituto Aragonés de Ciencias de la Salud; Osteba: Agencia de Evaluación de Tecnologías Sanitarias del País Vasco

[a]Published versions in GuiaSalud in March 2011.
