# Supplementary material for: Strategies to assess the validity of recommendations: a study protocol
Source: Implement Sci. 2013 Aug 22;8:94. doi: 10.1186/1748-5908-8-94 (PMC3765147; doi:10.1186/1748-5908-8-94)
Supplement: Additional file 2 — Recommendation baseline survey to clinical experts. Example on clinical practice guideline for secondary prevention of stroke (2009) [25]. [file 1748-5908-8-94-S2.pdf]

Additional file 2: Recommendation baseline survey to clinical experts. Example on Clinical Practice Guideline for Secondary Prevention of Stroke (2009)

**Key question**	In patients with a history of an episode of stroke, does antihypertensive treatment reduce the risk of new episodes?
**Recommendation**	Depending on the patient's tolerance or concomitant pathologies, monotherapy treatment with diuretics, angiotensin converting enzyme inhibitors or angiotensin II antagonists should be considered.
**Evidence quality (SIGN)**	1- to 1++
**Strength of recommendation (SIGN)**	B

1. Are you aware of new studies in the field relevant to this guideline recommendation?

☐ No

☐ Yes

☐ Don't know/no answer

2. If you are aware of this new evidence, are the new studies of sufficient importance to change the guideline recommendation?

☐ No

☐ Yes

☐ Don't know/no answer

If you are aware of these new published studies, please specify in each frame the citation of a study with one of the following formats:

- Title of the article. Journal title.
- Journal title. Year, volume (issue): initial-end page of the article.
- PMID ("PubMed Unique Identifier" unique number assigned to each PubMed citation of a biomedical journal article).
- Narrative comment if you do not remember the study (for example, the acronym of the study).

Study 1

Study 2

Study 3

Study 4

Study 5

