# Supplementary figures and images for: Strategies to assess the validity of recommendations: a study protocol
Source: Implement Sci. 2013 Aug 22;8:94. doi: 10.1186/1748-5908-8-94 (PMC3765147; doi:10.1186/1748-5908-8-94)

Additional File 3: Reference screening by pertinence

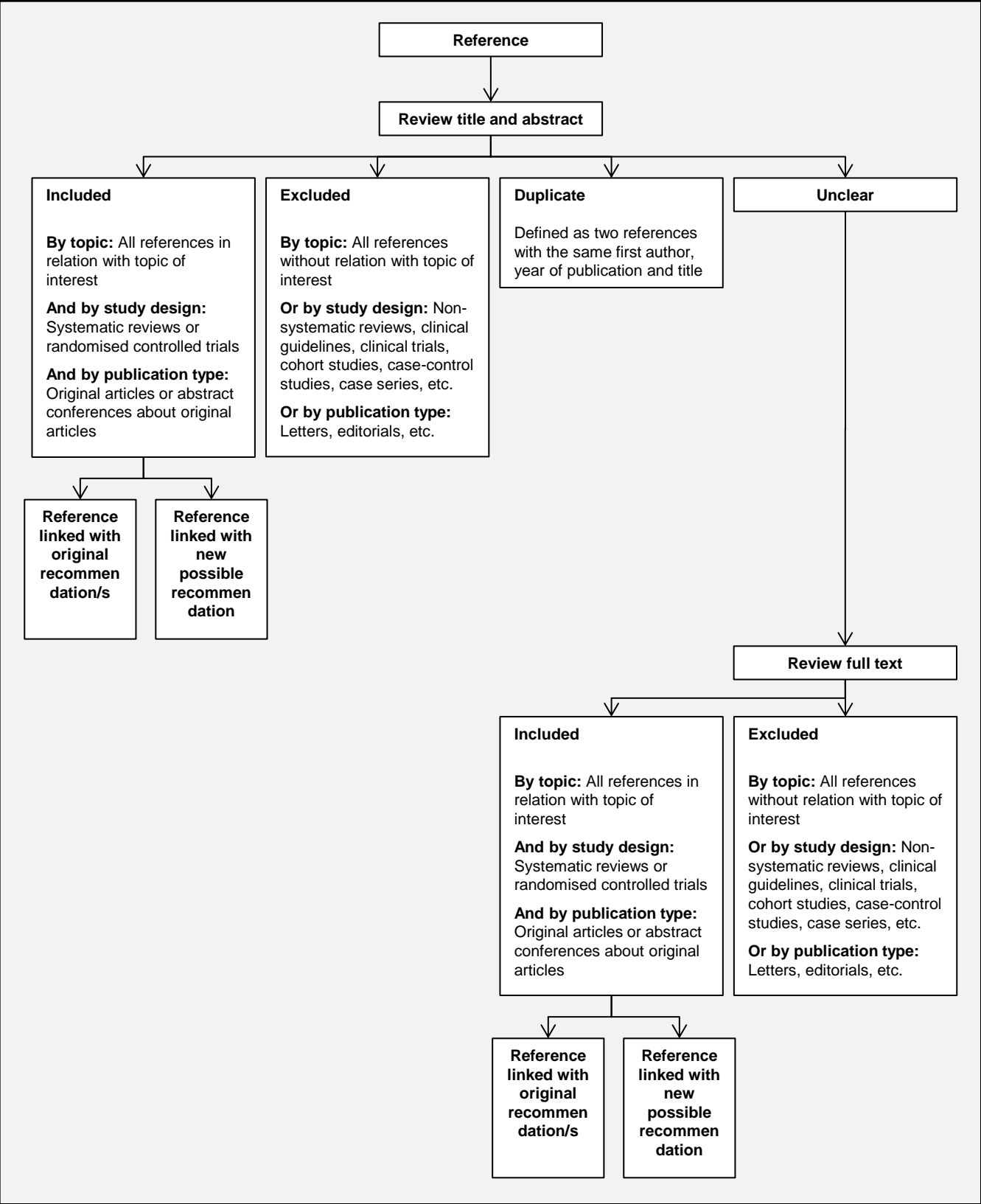

Supplement: Additional file 3 — Reference screening by pertinence. [file 1748-5908-8-94-S3.pdf]
