# Supplementary material for: Strategies to assess the validity of recommendations: a study protocol
Source: Implement Sci. 2013 Aug 22;8:94. doi: 10.1186/1748-5908-8-94 (PMC3765147; doi:10.1186/1748-5908-8-94)
Supplement: Additional file 4 — Reference survey to assess the updating effect. Example on clinical practice guideline for secondary prevention of stroke (2009) [25]. [file 1748-5908-8-94-S4.pdf]

Additional File 4: Reference survey to assess the updating effect. Example on Clinical Practice Guideline for Secondary Prevention of Stroke (2009)

**Key question**	In patients with a history of an episode of stroke, does antihypertensive treatment reduce the risk of new episodes?
**Recommendation**	Depending on the patient's tolerance or concomitant pathologies, monotherapy treatment with diuretics, angiotensin converting enzyme inhibitors or angiotensin II antagonists should be considered.
**Evidence quality (SIGN)**	1- to 1++
**Strength of recommendation (SIGN)**	B

**Reference 1**	Baker WL, White CM, Coleman CI. **The impact of angiotensin II receptor blocker potency on the clinical outcomes of stroke, acute myocardial infarction, or death.** Formulary 2007; 42(10):581-598.
**PMID**	-
**Abstract**	Angiotensin II receptor blockers (ARBs) have been demonstrated to reduce morbidity and/or mortality in patients with chronic heart failure (CHF), acute myocardial infarction (AMI), type 2 diabetes, and hypertension. Although as a class ARBs share a common mechanism of action, potency among the agents varies. Higher-potency ARBs (candesartan, irbesartan, olmesartan, and telmisartan) may demonstrate improved 24-hour blood pressure control, suggesting that these agents may have superior clinical event reduction potential versus lower-potency agents (eprosartan, losartan, and valsartan). We conducted a meta-analysis of randomized, controlled trials that evaluated the effect of ARBs on clinical outcomes. A systematic literature search of MEDLINE from 1966 through December 2006 was conducted using specific search terms. Studies that met the following criteria were included: randomized; not angiotensin-converting enzyme (ACE) inhibitor-controlled; incorporation of monotherapy with ARBs in ≥1 of the treatment arms; mean follow-up duration of ≥12 weeks; ≥ 100 patients enrolled; and data on primary or secondary stroke, AMI, or death reported. A randomeffects model was used for all meta-analyses conducted. Fourteen of the 6,641 citations initially identified met inclusion criteria, constituting a population of 46,874 patients. The ARBs, as a class, significantly reduced the incidence of stroke (OR=0.81; 95% Cl, 0.68-0.97) but had no effect on AMI or death. The high-potency ARBs significantly reduced the incidence of stroke (OR=0.78; 95% Cl, 0.66-0.91) but had no effect on AMI or death.The low-potency ARBs did not have a significant effect on stroke, AMI, or death. As we could not use indirect comparison techniques because of clinical heterogeneity, we cannot say with certainty that the high- and low-potency groups have different effects on clinical outcomes. A future meta-analysis should be conducted when other large clinical trials are published to determine if there is indeed a difference.
**Study design**	Systematic review

1. **Is this reference relevant to this recommendation (could be of use when considering updating this recommendation)?**
   - ☐ Yes. Please go to question 2.
   - ☐ No. Stop answering the questionnaire of this reference.
   - ☐ Doesn't know/answer. Stop answering the questionnaire of this reference.

2. **How does this reference affects the existing recommendation?**
   - ☐ Update of the recommendation needed (key reference). Please go to question 3.
   - ☐ Recommendation still valid (no update needed). Stop answering the questionnaire of this reference.
   - ☐ Doesn't know/answer. Stop answering the questionnaire of this reference.

3. **Given the reference above, do any of the following issues around this recommendation need to be modified? (possibility of more than one answer)**
   - ☐ The population
   - ☐ The intervention
   - ☐ The comparison
   - ☐ The outcome
   - ☐ The quality of the evidence
   - ☐ The direction of the recommendation
   - ☐ The strength of the recommendation
   - ☐ Doesn't know/answer

4. **Comments**

**Reference 2**	Brodszky V, Nagy V, Farsang C, Karpati K, Gulacsi L. **The efficacy of indapamide in different cardiovascular outcome--meta-analysis.** [Hungarian]. Orvosi hetilap 2007; 148(26):1203-1211.
**PMID**	17588853
**Abstract**	BACKGROUND: First line antihypertensive treatment's drugs have to be able to decrease the cardiovascular morbidity and mortality. This kind of efficacy of thiazides type diuretics were published earlier in several studies. The efficacy of indapamide was investigated in several studies, but there is no analysis which is including all of the indapamide-studies. OBJECTIVE: We conducted a meta-analysis of all relevant randomized-controlled-trials with indapamide. Efficacy of indapamide was analyzed in different cardiovascular and safety outcomes. METHODS: We searched the MEDLINE database 1995-2005 for indapamide-trials. Only double-blind, parallel-group design trials were involved. Both the fixed effect model and the random effect model were used for data synthesis, results were probed with Mantel-Hanzel test and inverse variance test. RESULTS: Data were combined from 9 trials that included 10 108 patients. Indapamide treatment of 48 patients with a history of stroke prevents another stroke (NNT = 47.8 95% CI 29.6-126.6). Data from 5 trials including 7085 patients show that indapamide is superior to placebo in reducing blood pressure, the differences are: 7.28 mm Hg (95% CI: 6.37-8.19) in systolic blood pressure and 3.50 mm Hg (95% CI 2.99-4.01) in diastolic blood pressure. Data from 5 trials including 2856 patients show that indapamide is superior to active controls in reducing systolic blood pressure, the difference is significant: 1.30 mm Hg (95% CI 0.28-2.31). The difference in diastolic blood pressure was not significant. Data of 505 patients show that indapamide reduced left ventricular mass index significantly more than enalapril 20 mg, the difference is 6.50 g/m(2) (95%CI: 0.81-12.19). Data of 6206 patients show that frequency of adverse drug reaction is similar in the indapamide and placebo groups (rr = 0.97 95%CI 0.76-1.22). CONCLUSIONS: Indapamide is efficacious in prevention of further stroke, reduces effectively the blood pressure and the left ventricular mass index. Indapamide treatment is well tolerated.
**Study design**	Systematic review

1. **Is this reference relevant to this recommendation (could be of use when considering updating this recommendation)?**
   - ☐ Yes. Please go to question 2.
   - ☐ No. Stop answering the questionnaire of this reference.
   - ☐ Doesn't know/answer. Stop answering the questionnaire of this reference.

2. **How does this reference affects the existing recommendation?**

☐ Update of the recommendation needed (key reference). Please go to question 3.
☐ Recommendation still valid (no update needed). Stop answering the questionnaire of this reference.
☐ Doesn't know/answer. Stop answering the questionnaire of this reference.

3. **Given the reference above, do any of the following issues around this recommendation need to be modified? (possibility of more than one answer)**

☐ The population
☐ The intervention
☐ The comparison
☐ The outcome
☐ The quality of the evidence
☐ The direction of the recommendation
☐ The strength of the recommendation
☐ Doesn't know/answer

4. **Comments**

**Reference 3**	Lee MS, Pittler MH, Guo R, Ernst E. **Qigong for hypertension: A systematic review of randomized clinical trials.** Journal of Hypertension 2007; 25(8):1525-1532.
**PMID**	17620944
**Abstract**	OBJECTIVES: To assess systematically the clinical evidence of qigong for hypertension. METHODS: Databases were searched up to August 2006. All randomized clinical trials (RCTs) testing qigong in patients with hypertension of any origin and assessing clinically relevant outcomes were considered. Trials using any type of control intervention were included. The selection of studies, data extraction and quality assessment were performed independently by at least two reviewers. Methodological quality was evaluated using the Jadad score. RESULTS: A total of 121 potentially relevant articles were identified and 12 RCTs were included. Seven RCTs tested qigong in combination with antihypertensive drugs compared with antihypertensive drugs alone. The meta-analysis of two trials reporting adequate data suggested beneficial effects in favour of qigong [weighted mean difference, systolic blood pressure (SBP) -12.1 mmHg, 95% confidence interval (CI) -17.1 to -7.0; diastolic blood pressure -8.5 mmHg, 95% CI -12.6 to -4.4]. Qigong was compared with waiting list control in two RCTs and was found to reduce SBP significantly (weighted mean difference -18.5 mmHg, 95% CI -23.1 to -13.9). In three further RCTs the comparisons made were: qigong combined with conventional therapy versus muscle relaxation combined with conventional therapy; qigong as a sole treatment versus exercise. All reported positive results in at least some of the relevant outcome measures. The methodological quality of the studies was low. CONCLUSION: There is some encouraging evidence of qigong for lowering SBP, but the conclusiveness of these findings is limited. Rigorously designed trials are warranted to confirm these results.
**Study design**	Systematic review

1. **Is this reference relevant to this recommendation (could be of use when considering updating this recommendation)?**

☐ Yes. Please go to question 2.
☐ No. Stop answering the questionnaire of this reference.
☐ Doesn't know/answer. Stop answering the questionnaire of this reference.

2. **How does this reference affects the existing recommendation?**

☐ Update of the recommendation needed (key reference). Please go to question 3.
☐ Recommendation still valid (no update needed). Stop answering the questionnaire of this reference.
☐ Doesn't know/answer. Stop answering the questionnaire of this reference.

3. **Given the reference above, do any of the following issues around this recommendation need to be modified? (possibility of more than one answer)**
☐ The population
☐ The intervention
☐ The comparison
☐ The outcome
☐ The quality of the evidence
☐ The direction of the recommendation
☐ The strength of the recommendation
☐ Doesn't know/answer

4. **Comments**

**Reference 4**	Saha SA, Molnar J, Arora RR. **Tissue ACE inhibitors for secondary prevention of cardiovascular disease in patients with preserved left ventricular function: a pooled meta-analysis of randomized placebo-controlled trials.** J Cardiovasc Pharmacol Ther 2007; 12(3):192-204.
**PMID**	17875946
**Abstract**	OBJECTIVE: A pooled meta-analysis of published, randomized placebo-controlled clinical trials to evaluate the role of tissue angiotensin-converting enzyme (ACE) inhibitors in secondary prevention of cardiovascular disease in patients with preserved left ventricular function. SOURCES: Peer-reviewed journals listed in Index Medicus/MEDLINE, the Cochrane Central Register of Controlled Clinical Trials, and the Cochrane Database of Systematic Reviews. STUDY SELECTION: Randomized placebo-controlled clinical trials of at least 12 months' duration, in patients with a prior cardiovascular event or at high risk for cardiovascular events, were analyzed. DATA SYNTHESIS AND ANALYSIS: A total of 31,555 patients (136,882 patient-years) from 4 trials were selected for the meta-analysis. Relative risk estimations were made using data pooled from these trials, and statistical significance was determined using the chi2 test. The number of patients needed to treat was also calculated for each outcome. RESULTS: Tissue ACE inhibitors significantly reduced the risk of all-cause mortality, cardiovascular mortality, acute myocardial infarction, and stroke (P < .001 for each). The need for invasive coronary revascularization was reduced (P = .03), as was the risk of hospitalization for congestive heart failure (P = .001). The occurrence of new-onset diabetes was also significantly reduced (P < .001), but the risk of hospitalization for angina was not significantly affected (P = .677). Treating about 100 patients for about 4.5 years would prevent 1 death, 1 non-fatal myocardial infarction, 1 cardiovascular death, or 1 invasive coronary revascularization. CONCLUSIONS: Tissue ACE inhibitors have demonstrated benefit when used for secondary prevention of cardiovascular disease in patients with preserved left ventricular function in randomized placebo-controlled clinical trials.
**Study design**	Systematic review

1. **Is this reference relevant to this recommendation (could be of use when considering updating this recommendation)?**
☐ Yes. Please go to question 2.
☐ No. Stop answering the questionnaire of this reference.

☐ Doesn't know/answer. Stop answering the questionnaire of this reference.

2. **How does this reference affects the existing recommendation?**
   ☐ Update of the recommendation needed (key reference). Please go to question 3.
   ☐ Recommendation still valid (no update needed). Stop answering the questionnaire of this reference.
   ☐ Doesn't know/answer. Stop answering the questionnaire of this reference.

3. **Given the reference above, do any of the following issues around this recommendation need to be modified? (possibility of more than one answer)**
   ☐ The population
   ☐ The intervention
   ☐ The comparison
   ☐ The outcome
   ☐ The quality of the evidence
   ☐ The direction of the recommendation
   ☐ The strength of the recommendation
   ☐ Doesn't know/answer

4. **Comments**

**Reference 5**	Bath PM, Martin RH, Palesch Y, Cotton D, Yusuf S, Sacco R et al. **Effect of telmisartan on functional outcome, recurrence and blood pressure in patients with acute ischaemic stroke: A PRoFESS substudy.** Stroke Conference: 2009.
**PMID**	-
**Abstract**	Introduction: High blood pressure is common in acute ischaemic stroke and associated with a poor functional outcome. However, the management of high BP remains unclear and no large trials have been completed. Methods: The PRoFESS trial evaluated the effects of lowering BP with telmisartan (angiotensin receptor antagonist) in patients with ischaemic stroke; patients were also randomised to aspirin/extended release dipyridamole vs. clopidogrel in a factorial design. Of 20,332 patients, 1,360 were randomised to telmisartan (80 mg/day, n=647) or matching placebo (n=713) within 72 hours of onset. The primary outcome was functional outcome; secondary outcomes included death, recurrence and haemodynamic measures. Analyses were adjusted for baseline prognostic variables (age, sex, NIH stroke scale, systolic BP), non-trial antihypertensive agents, and antiplatelet group. Results: Patients were representative of the whole trial (age 67 years, male 65%, BP 147/84 mmHg, small artery occlusion 60%) and baseline variables were matched between treatment groups. The mean time to recruitment was 58 hours. Combined death or dependency (modified Rankin Scale >1, 30 days, OR 1.00, 95% CI 0.78-1.31), death (90 days, OR 0.88, 95% CI 0.23-3.47) and recurrence (90 days, OR 1.37, 95% CI 0.66-2.82) did not differ between the treatment groups. In comparison with placebo, telmisartan lowered BP by 6-7/2-4 mmHg over the first 90 days (p<0.001), pulse pressure (3-4 mmHg), and rate-pressure product (466 mmHg.bpm); no effect on heart rate was seen. Conclusion: This substudy of PRoFESS is the largest (1,360 patients) randomised assessment of BP lowering in patients with acute ischaemic stroke. Treatment with telmisartan lowered BP but did not alter functional dependency, death or recurrence by 90 days post stroke. However, the mean BP was only moderately elevated and the sample size was relatively small in comparison with ongoing trials of BP management in acute stroke (range 2,500-5,000).
**Study design**	Randomized control trial

1. **Is this reference relevant to this recommendation (could be of use when considering updating this recommendation)?**
   - ☐ Yes. Please go to question 2.
   - ☐ No. Stop answering the questionnaire of this reference.
   - ☐ Doesn't know/answer. Stop answering the questionnaire of this reference.

2. **How does this reference affects the existing recommendation?**
   - ☐ Update of the recommendation needed (key reference). Please go to question 3.
   - ☐ Recommendation still valid (no update needed). Stop answering the questionnaire of this reference.
   - ☐ Doesn't know/answer. Stop answering the questionnaire of this reference.

3. **Given the reference above, do any of the following issues around this recommendation need to be modified? (possibility of more than one answer)**
   - ☐ The population
   - ☐ The intervention
   - ☐ The comparison
   - ☐ The outcome
   - ☐ The quality of the evidence
   - ☐ The direction of the recommendation
   - ☐ The strength of the recommendation
   - ☐ Doesn't know/answer

4. **Comments**

**Reference 6**	Costanzo P, Perrone-Filardi P, Petretta M, Marciano C, Gargiulo P, Guerra G et al. **Calcium channel blockers and cardiovascular outcomes: A meta-analysis.** Journal of the American College of Cardiology Conference: American College of Cardiology 2009; 58th Annual Scientific Session: Innovation.
**PMID**	-
**Abstract**	Background: The aim of this study was to update the previous meta-analyses with the results of the recent trials, assessing the effect of CCBs treatment, compared to other drugs or placebo/top of therapy, on all cause mortality, cardiovascular death, major cardiovascular events, heart failure, myocardial infarction and stroke. Methods: We performed a meta-analysis of randomized controlled trials that compared a long acting calcium channel blocker with another drug or placebo/top of therapy and that assessed all cause mortality and cardiovascular events. Overall estimates of effect were calculated with a fixed-effects, random effects model or Peto method. We performed also random effects meta-regression analysis to assess the influence of blood pressure reduction on the outcomes. Results: We included 27 trials (175,634 patients). The risk of all cause death was reduced by dihydropyridine CCBs (OR 0.96, 95% CI 0.93 to 0.99, p = 0.03; heterogeneity (h) p = 0.87) without influence of placebo trials and blood pressure reduction. The risk of heart failure was increased by CCBs compared to active treatment (OR 1.17, 95% CI 1.11 to 1.24, p = 0.0001; h p = 0.0001) and it was decreased when compared to placebo (OR 0.72, 95% CI 0.59 to 0.87, p = 0.001; h p = 0.77), also in the subgroup of coronary artery disease patients (OR 0.76, 95% CI 0.61 to 0.95, p = 0.01; h p = 0.29). CCBs did not increase the risk of myocardial infarction (OR 1, 95% CI 0.95 to 1.04, p = 0.83; h p = 0.004), cardiovascular death (OR 0.97, 95% CI 0.93 to 1.02, p = 0.24; h p = 0.16), major cardiovascular events (OR 0.97, 95% CI 0.90 to 1.06, p = 0.53; h p = 0.0001). CCBs decreased the risk of fatal or nonfatal stroke (OR 0.86, 95% CI 0.82 to

	0.90, comparison p = 0.0001; h p = 0.12), also when compared to ACE inhibitors (OR 0.87, 95% CI 0.78 to 0.97, p = 0.016; h p = 0.48) Conclusions: Our study demonstrates that calcium antagonists reduce the risk of all cause mortality compared to active therapy, and prevent heart failure compared to placebo. Furthermore, with the inclusion of recent trials, we confirm that they reduce the risk of stroke, also in comparison to ace inhibitors and do not increase the risk of cardiovascular death, myocardial infarction and major cardiovascular events.
**Study design**	Systematic review

1. **Is this reference relevant to this recommendation (could be of use when considering updating this recommendation)?**
   ☐ Yes. Please go to question 2.
   ☐ No. Stop answering the questionnaire of this reference.
   ☐ Doesn't know/answer. Stop answering the questionnaire of this reference.

2. **How does this reference affects the existing recommendation?**
   ☐ Update of the recommendation needed (key reference). Please go to question 3.
   ☐ Recommendation still valid (no update needed). Stop answering the questionnaire of this reference.
   ☐ Doesn't know/answer. Stop answering the questionnaire of this reference.

3. **Given the reference above, do any of the following issues around this recommendation need to be modified? (possibility of more than one answer)**
   ☐ The population
   ☐ The intervention
   ☐ The comparison
   ☐ The outcome
   ☐ The quality of the evidence
   ☐ The direction of the recommendation
   ☐ The strength of the recommendation
   ☐ Doesn't know/answer

4. **Comments**

**Reference 7**	Lakhan SE, Sapko MT. **Blood pressure lowering treatment for preventing stroke recurrence: a systematic review and meta-analysis.** Int Arch Med 2009; 2(1):30.
**PMID**	19843330
**Abstract**	BACKGROUND: While hypertension is a leading risk factor for an initial stroke, the role of blood pressure lowering to prevent subsequent stroke is less clear. The results of recent large clinical trials investigating effects of antihypertensive agents in patients with a history of stroke have not shown a significant benefit; findings that are at odds with previous data. Our meta-analysis systematically evaluates the available, relevant trials to examine the role of antihypertensive drugs in preventing recurrent stroke. METHODS: MEDLINE, CENTRAL, and ClinicalTrials.gov were systematically searched and bibliographies from key reports were examined. All randomized, placebo-controlled trials that tested blood pressure lowering agents in patients with stroke or transient ischemic attack were identified. The results from these trials were combined and meta-analyses were performed. RESULTS: Ten studies were found to contain relevant endpoints and presented data allowing meta-analysis. Agents that lowered blood pressure reduced recurrent stroke (OR 0.71, 95% CI

	0.59-0.86, P = 0.0004) and cardiovascular events (OR 0.69, 95% CI 0.57-0.85, P = 0.0004) in patients with a previous stroke or TIA. These agents did not affect the rate of myocardial infarction (OR 0.86, 95% CI 0.73-1.01, P = 0.07) or all-cause mortality (OR 0.95, 95% CI 0.83-1.07, P = 0.39) in this patient population. CONCLUSION: Despite recent large trials showing no significant effect, in patients that have experienced a TIA or stroke, blood pressure lowering agents reduced the occurrence of subsequent stroke and cardiovascular events. The rate of myocardial infarction and all-cause mortality was unchanged.
**Study design**	Systematic review

1. **Is this reference relevant to this recommendation (could be of use when considering updating this recommendation)?**
   - ☐ Yes. Please go to question 2.
   - ☐ No. Stop answering the questionnaire of this reference.
   - ☐ Doesn't know/answer. Stop answering the questionnaire of this reference.

2. **How does this reference affects the existing recommendation?**
   - ☐ Update of the recommendation needed (key reference). Please go to question 3.
   - ☐ Recommendation still valid (no update needed). Stop answering the questionnaire of this reference.
   - ☐ Doesn't know/answer. Stop answering the questionnaire of this reference.

3. **Given the reference above, do any of the following issues around this recommendation need to be modified? (possibility of more than one answer)**
   - ☐ The population
   - ☐ The intervention
   - ☐ The comparison
   - ☐ The outcome
   - ☐ The quality of the evidence
   - ☐ The direction of the recommendation
   - ☐ The strength of the recommendation
   - ☐ Doesn't know/answer

4. **Comments**

**Reference 8**	Law MR, Morris JK, Wald NJ. **Use of blood pressure lowering drugs in the prevention of cardiovascular disease: meta-analysis of 147 randomised trials in the context of expectations from prospective epidemiological studies.** BMJ 2009; 338:b1665.
**PMID**	19454737
**Abstract**	OBJECTIVES: To determine the quantitative efficacy of different classes of blood pressure lowering drugs in preventing coronary heart disease (CHD) and stroke, and who should receive treatment. DESIGN: Meta-analysis. Data source Medline (1966-2007). STUDY SELECTION: Randomised trials of blood pressure lowering drugs recording CHD events and strokes. 108 trials studied differences in blood pressure between study drug and placebo (or control group not receiving the study drug) ("blood pressure difference trials"), and 46 trials compared drugs ("drug comparison trials"). Seven trials with three randomised groups fell into both categories. The results were interpreted in the context of those expected from the largest published meta-

	analysis of cohort studies, totalling 958 000 people. PARTICIPANTS: 464 000 people defined into three mutually exclusive categories: participants with no history of vascular disease, a history of CHD, or a history of stroke. RESULTS: In the blood pressure difference trials beta blockers had a special effect over and above that due to blood pressure reduction in preventing recurrent CHD events in people with a history of CHD: risk reduction 29% (95% confidence interval 22% to 34%) compared with 15% (11% to 19%) in trials of other drugs. The extra effect was limited to a few years after myocardial infarction, with a risk reduction of 31% compared with 13% in people with CHD with no recent infarct (P=0.04). In the other blood pressure difference trials (excluding CHD events in trials of beta blockers in people with CHD), there was a 22% reduction in CHD events (17% to 27%) and a 41% (33% to 48%) reduction in stroke for a blood pressure reduction of 10 mm Hg systolic or 5 mm Hg diastolic, similar to the reductions of 25% (CHD) and 36% (stroke) expected for the same difference in blood pressure from the cohort study meta-analysis, indicating that the benefit is explained by blood pressure reduction itself. The five main classes of blood pressure lowering drugs (thiazides, beta blockers, angiotensin converting enzyme inhibitors, angiotensin receptor blockers, and calcium channel blockers) were similarly effective (within a few percentage points) in preventing CHD events and strokes, with the exception that calcium channel blockers had a greater preventive effect on stroke (relative risk 0.92, 95% confidence interval 0.85 to 0.98). The percentage reductions in CHD events and stroke were similar in people with and without cardiovascular disease and regardless of blood pressure before treatment (down to 110 mm Hg systolic and 70 mm Hg diastolic). Combining our results with those from two other studies (the meta-analyses of blood pressure cohort studies and of trials determining the blood pressure lowering effects of drugs according to dose) showed that in people aged 60-69 with a diastolic blood pressure before treatment of 90 mm Hg, three drugs at half standard dose in combination reduced the risk of CHD by an estimated 46% and of stroke by 62%; one drug at standard dose had about half this effect. The present meta-analysis also showed that drugs other than calcium channel blockers (with the exception of non-cardioselective beta blockers) reduced the incidence of heart failure by 24% (19% to 28%) and calcium channel blockers by 19% (6% to 31%). CONCLUSIONS: With the exception of the extra protective effect of beta blockers given shortly after a myocardial infarction and the minor additional effect of calcium channel blockers in preventing stroke, all the classes of blood pressure lowering drugs have a similar effect in reducing CHD events and stroke for a given reduction in blood pressure so excluding material pleiotropic effects. The proportional reduction in cardiovascular disease events was the same or similar regardless of pretreatment blood pressure and the presence or absence of existing cardiovascular disease. Guidelines on the use of blood pressure lowering drugs can be simplified so that drugs are offered to people with all levels of blood pressure. Our results indicate the importance of lowering blood pressure in everyone over a certain age, rather than measuring it in everyone and treating it in some.
**Study design**	Systematic review

1. **Is this reference relevant to this recommendation (could be of use when considering updating this recommendation)?**
   ☐ Yes. Please go to question 2.
   ☐ No. Stop answering the questionnaire of this reference.
   ☐ Doesn't know/answer. Stop answering the questionnaire of this reference.

2. **How does this reference affects the existing recommendation?**
   ☐ Update of the recommendation needed (key reference). Please go to question 3.
   ☐ Recommendation still valid (no update needed). Stop answering the questionnaire of this reference.
   ☐ Doesn't know/answer. Stop answering the questionnaire of this reference.

3. **Given the reference above, do any of the following issues around this recommendation need to be modified? (possibility of more than one answer)**
   ☐ The population
   ☐ The intervention
   ☐ The comparison

☐ The outcome

☐ The quality of the evidence

☐ The direction of the recommendation

☐ The strength of the recommendation

☐ Doesn't know/answer

4. Comments

**Reference 9**	Lu GC, Cheng JW, Zhu KM, Ma XJ, Shen FM, Su DF. **A systematic review of angiotensin receptor blockers in preventing stroke.** Stroke 2009; 40(12):3876-3878.
**PMID**	19892999
**Abstract**	BACKGROUND AND PURPOSE: Angiotensin receptor blockers are widely used in patients at high risk of cardiocerebrovascular events. The aim of this meta-analysis was to investigate the effects of angiotensin receptor blockers on the risk of stroke. METHODS: Electronic searches of MEDLINE, EMBASE, and the Cochrane central register of controlled trials were performed. A total of 20 randomized clinical trials with 108 286 patients reporting stroke were available for this clinical outcome analysis. RESULTS: Angiotensin receptor blockers were associated with a significant reduction in the risk of stroke than placebo with an OR of 0.91 (0.84 to 0.98). Angiotensin receptor blockers were associated with no significant reduction in the risk of stroke compared with angiotensin-converting enzyme inhibitors (OR, 0.93; 0.84 to 1.03) and calcium antagonists (OR, 1.16; 0.91 to 1.48). CONCLUSIONS: Evidence of the benefit of angiotensin receptor blockers on the risk of stroke is provided when compared with placebo. There was no evidence of the benefit when comparing angiotensin receptor blockers with angiotensin-converting enzyme inhibitors and with calcium antagonists.
**Study design**	Systematic review

1. Is this reference relevant to this recommendation (could be of use when considering updating this recommendation)?

☐ Yes. Please go to question 2.

☐ No. Stop answering the questionnaire of this reference.

☐ Doesn't know/answer. Stop answering the questionnaire of this reference.

2. How does this reference affects the existing recommendation?

☐ Update of the recommendation needed (key reference). Please go to question 3.

☐ Recommendation still valid (no update needed). Stop answering the questionnaire of this reference.

☐ Doesn't know/answer. Stop answering the questionnaire of this reference.

3. Given the reference above, do any of the following issues around this recommendation need to be modified? (possibility of more than one answer)

☐ The population

☐ The intervention

☐ The comparison

☐ The outcome

☐ The quality of the evidence

☐ The direction of the recommendation

☐ The strength of the recommendation

☐ Doesn't know/answer

4. **Comments**

**Reference 10**	Talbert RL. **Role of antihypertensive therapy with angiotensin-converting enzyme inhibitors or angiotensin II receptor blockers in combination with calcium channel blockers for stroke prevention.** J Am Pharm Assoc (2003) 2010; 50(5):e116-e125.
**PMID**	20833609
**Abstract**	OBJECTIVE: To review the available literature on the effects of angiotensin-converting enzyme inhibitors (ACEIs), angiotensin II receptor blockers (ARBs), and calcium channel blockers (CCBs) or combinations of these agents on stroke outcomes in hypertensive patients. DATA SOURCES: A Medline search was conducted using the search terms stroke and antihypertensives, calcium channel blockers, angiotensin-converting enzyme inhibitors, or angiotensin II receptor blockers from 1985 to August 17, 2009. STUDY SELECTION: Randomized controlled clinical trials with at least 400 randomized patients were selected if at least one of the treatment arms used a CCB, ACEI, or ARB to evaluate stroke outcomes in hypertensive patients. DATA SYNTHESIS: The prevalence of stroke is high in the United States, accounting for approximately 150,000 deaths per year. Early identification and treatment of hypertension to quickly achieve blood pressure reduction is critical in the prevention of stroke. Many trials have provided evidence that CCBs, ACEIs, and ARBs are effective in stroke prevention. Most patients require two or more antihypertensive drugs to achieve blood pressure goals. Because of their complementary actions, combination antihypertensive therapy with a renin-angiotensin-aldosterone system (RAAS) blocker and a CCB may help reduce stroke incidence to a greater extent than either of the monotherapies. CONCLUSION: A growing body of clinical trial data suggest that aggressive combination antihypertensive therapy, including a RAAS blocker and CCB, may help reduce stroke incidence. Fixed-dose combination therapy is an important consideration in optimizing blood pressure control and patient adherence to therapy in stroke prevention.consideration in optimizing blood pressure control and patient adherence to therapy in stroke prevention
**Study design**	Systematic review

1. **Is this reference relevant to this recommendation (could be of use when considering updating this recommendation)?**

   ☐ Yes. Please go to question 2.
   ☐ No. Stop answering the questionnaire of this reference.
   ☐ Doesn't know/answer. Stop answering the questionnaire of this reference.

2. **How does this reference affects the existing recommendation?**

   ☐ Update of the recommendation needed (key reference). Please go to question 3.
   ☐ Recommendation still valid (no update needed). Stop answering the questionnaire of this reference.
   ☐ Doesn't know/answer. Stop answering the questionnaire of this reference.

3. **Given the reference above, do any of the following issues around this recommendation need to be modified? (possibility of more than one answer)**

   ☐ The population
   ☐ The intervention
   ☐ The comparison
   ☐ The outcome
   ☐ The quality of the evidence

☐ The direction of the recommendation

☐ The strength of the recommendation

☐ Doesn't know/answer

4. **Comments**

**Reference 11**	Webb A, Rothwell PM, Fischer U, Mehta Z. **Drug-class effects on consistency of control of blood pressure and hence on stroke risk: Systematic review of 1372 trials.** Journal of Neurology, Neurosurgery and Psychiatry Conference: Association of British Neurologists Annual Meeting 2010.
**PMID**	-
**Abstract**	Introduction: Classes of blood pressure lowering drugs (BPLD) result in unexplained differences in stroke risk, independent of mean BP. We did a systematic review of BPLD on consistency of control of BP in randomised controlled trials (RCTs), related to stroke risk. Methods: Baseline and follow-up mean (SD) BP were extracted from all relevant RCTs. Effect of BPLD on variance in BP (SD-squared) was expressed as the variance ratio (VR), by random-effects meta-analysis. In trials with ≥100 patient-years per treatment group, VR was correlated with stroke risk, adjusted for mean BP. Results: Of 1372 eligible trials, 389 reported mean BP and SD at follow-up. Of the substantial heterogeneity between trials in VR ($p<1\times10^{-40}$), 68% was due to drug class. Compared to other drugs, SD-SBP was lower on calcium antagonists (VR=0.81 95% CI 0.76 to 0.86, p<0.001) and diuretics (0.87, 0.79–0.96, p=0.007), and higher on ACE-inhibitors (1.08, 1.02–1.15, p=0.008), ARBs (1.16, 1.07–1.25, p<0.001) and β-blockers (1.17, 1.07–1.28, p<0.001). Across all classes, reduced VR was associated with a reduced risk of stroke (VR<1.0-OR 0.87, 0.78–0.97, p=0.012; VR≤0.80-OR 0.79, 0.71–0.87, p<0.0001), which was significant (p=0.0016) when adjusted for change in mean BP. Conclusion: Substantial drug-class effects on consistency of control of BP, independent of mean, account for differences in stroke risk. Consistency of control of SBP is as important as extent of control (mean BP) in preventing stroke.
**Study design**	Systematic review

1. **Is this reference relevant to this recommendation (could be of use when considering updating this recommendation)?**

☐ Yes.	Please go to question 2.
☐ No.	Stop answering the questionnaire of this reference.
☐ Doesn't know/answer.	Stop answering the questionnaire of this reference.

2. **How does this reference affects the existing recommendation?**

☐ Update of the recommendation needed (key reference).	Please go to question 3.
☐ Recommendation still valid (no update needed).	Stop answering the questionnaire of this reference.
☐ Doesn't know/answer.	Stop answering the questionnaire of this reference.

3. **Given the reference above, do any of the following issues around this recommendation need to be modified? (possibility of more than one answer)**

☐ The population

☐ The intervention

☐ The comparison

☐ The outcome

☐ The quality of the evidence

☐ The direction of the recommendation

☐ The strength of the recommendation

☐ Doesn't know/answer

4. Comments

**Reference 12**	Lee M, Saver JL, Hong KS, Hao Q, Chow J, Ovbiagele B. **Angiotensin converting enzyme inhibitors or angiotensin receptor blockers reduce future vascular risk in persons with a history of stroke: A meta-analysis.** Stroke Conference: 2011.
**PMID**	-
**Abstract**	Background: Randomized clinical trials have shown that thiazide-based regimens reduce vascular risk in persons with known stroke. Although presumed efficacious, the impact of non-diuretic-based antihypertensives on vascular outcomes after stroke has been less well studied and is not convincingly proven. Specifically the totality of evidence regarding efficacy of renin angiotensin system (RAS) modulators in individuals with prior stroke is unclear. We assessed the efficacy of RAS modulators in persons with a history of stroke by conducting a systematic review and meta-analysis. Methods: Systematic literature search was performed. Inclusion criteria were 1) a randomized controlled trial; 2) participants with stroke history; 3) angiotensin converting enzyme (ACE) inhibitors or angiotensin receptor blockers (ARB) as mandatory therapy in the active treatment group; 4) trials reporting cardiovascular events or just recurrent stroke as outcomes; 5) follow-up duration of at least 6 months. Studies were excluded if 1) mandatory ACE inhibitor or ARB used in control group; 2) additional treatment besides ACE inhibitor or ARB used in active group. Results: The search identified 7 randomized controlled trials comprising 28,805 participants with a history of stroke. Across all trials, RAS modulator therapy reduced cardiovascular events (relative risk 0.91, 95% confidence interval 0.86 to 0.96, P<0.001) (Fig 1). Across 6 trials with 25,791 participants with recurrent stroke reported as an endpoint, RAS modulator therapy marginally reduced stroke (relative risk 0.93, 95% CI confidence interval 0.86 to 1.00, P=0.05) (Fig 2). Heterogeneity existed between estimates of cardiovascular events (p<0.01, I² =65%) but not estimates of recurrent stroke (P²0.46, I² =0%). Conclusions: ACE inhibitor or ARB therapy modestly reduces the risk of future cardiovascular events in persons with a history of stroke. ACE inhibitor or ARB could be considered in patients with a history of stroke as add-on therapy, or in thiazide-intolerant patients. (Table presented)
**Study design**	Systematic review

1. Is this reference relevant to this recommendation (could be of use when considering updating this recommendation)?

☐ Yes. Please go to question 2.

☐ No. Stop answering the questionnaire of this reference.

☐ Doesn't know/answer. Stop answering the questionnaire of this reference.

2. How does this reference affects the existing recommendation?

☐ Update of the recommendation needed (key reference). Please go to question 3.

☐ Recommendation still valid (no update needed). Stop answering the questionnaire of this reference.

☐ Doesn't know/answer. Stop answering the questionnaire of this reference.

3. Given the reference above, do any of the following issues around this recommendation need to be modified? (possibility of more than one answer)

☐ The population

☐ The intervention

☐ The comparison

☐ The outcome

☐ The quality of the evidence

☐ The direction of the recommendation

☐ The strength of the recommendation

☐ Doesn't know/answer

4. **Comments**

**Reference 13**	Wang Z, Chen Z, Richart T, Staessen J, Liu L. **Blood pressure lowering for the prevention of stroke recurrence.** International Journal of Cardiology Conference: World Hypertension League Regional Congress 2011.
**PMID**	-
**Abstract**	Objective: To investigate the effects of antihypertensive drug treatment on recurrent stroke in patients with a previous history of cerebrovascular disease. Methods: Original research papers published between 2000 and 2009, which focused on prevention of stroke recurrence by lowering blood pressure, were searched through PubMed database. The qualified data were processed with a meta-analysis. Data from trials that used a diuretic as opposed to a renin system inhibitors as themainstay of active treatmentwere compared for subgroup analyses. Results: Ten randomized controlled trials were eligible for analysis, including 38,227 patients together. The pooled odds ratio for the prevention was 0.78 (95% confidence interval (CI), 0.68 to 0.90; P=0.007), 0.63 (CI, 0.54 to 0.73; P<0.0001) for trials involving a diuretic as a component of experimental therapy, and 0.93 (CI, 0.87 to 1.01; P=0.086) in the trials inwhich the mainstay of treatment consisted of inhibition of the renin system. The P-value for heterogeneity between studies involving diuretics as compared with renin system inhibitors was less than 0.0001. There was also heterogeneity in the odds ratios among all trials (P<0.0001). The blood pressure reduction averaged 5.1/2.5 mmHg of systolic/diastolic in all studies, 8.5/3.6mmHg in the trials of diuretics, 4.0 /2.1 mmHg in the studies of renin system inhibitors. The correlation between the odds of stroke recurrence and the reduction in systolic blood pressure approached significance. Conclusions: Blood pressure lowering reduced the risk of recurrent stroke significantly
**Study design**	Systematic review

1. **Is this reference relevant to this recommendation (could be of use when considering updating this recommendation)?**

☐ Yes. Please go to question 2.

☐ No. Stop answering the questionnaire of this reference.

☐ Doesn't know/answer. Stop answering the questionnaire of this reference.

2. **How does this reference affects the existing recommendation?**

☐ Update of the recommendation needed (key reference). Please go to question 3.

☐ Recommendation still valid (no update needed). Stop answering the questionnaire of this reference.

☐ Doesn't know/answer. Stop answering the questionnaire of this reference.

3. **Given the reference above, do any of the following issues around this recommendation need to be modified? (possibility of more than one answer)**

☐ The population

- ☐ The intervention
- ☐ The comparison
- ☐ The outcome
- ☐ The quality of the evidence
- ☐ The direction of the recommendation
- ☐ The strength of the recommendation
- ☐ Doesn't know/answer

4. Comments

